# Nicotine Exacerbates Arrhythmogenesis in Rabbit Right Ventricular Outflow Tract Triggered by Chronic Obstructive Pulmonary Disease

**DOI:** 10.1111/jcmm.70664

**Published:** 2025-06-18

**Authors:** Chao‐Shun Chan, Feng‐Zhi Lin, Yao‐Chang Chen, Satoshi Higa, Shih‐Ann Chen, Yi‐Jen Chen

**Affiliations:** ^1^ Division of Cardiology, Department of Internal Medicine, School of Medicine, College of Medicine Taipei Medical University Taipei Taiwan; ^2^ Division of Cardiology, Department of Internal Medicine Taipei Medical University Hospital, Taipei Medical University Taipei Taiwan; ^3^ Graduate Institute of Medical Sciences, College of Medicine Taipei Medical University Taipei Taiwan; ^4^ Department of Biomedical Engineering National Defense Medical Center Taipei Taiwan; ^5^ Cardiac Electrophysiology and Pacing Laboratory, Division of Cardiovascular Medicine Makiminato Central Hospital Okinawa Japan; ^6^ Heart Rhythm Center, Division of Cardiology, Department of Medicine Taipei Veterans General Hospital Taipei Taiwan; ^7^ Institute of Clinical Medicine and Faculty of Medicine National Yang Ming Chiao Tung University Taipei Taiwan; ^8^ Cardiovascular Center Taichung Veterans General Hospital Taichung Taiwan; ^9^ Division of Cardiology, Department of Internal Medicine, Wan‐Fang Hospital Taipei Medical University Taipei Taiwan; ^10^ Graduate Institute of Clinical Medicine, College of Medicine Taipei Medical University Taipei Taiwan; ^11^ Cardiovascular Research Center, Wan‐Fang Hospital Taipei Medical University Taipei Taiwan

**Keywords:** chronic obstructive pulmonary disease, nicotine, right ventricular outflow tract, ventricular tachycardia

## Abstract

Cigarette smoke includes nicotine that increases ventricular tachycardia (VT) risk. Chronic obstructive pulmonary disease (COPD) and right ventricular outflow tract (RVOT) constitute the primary risk factor and origin of VT, respectively. To investigate the arrhythmogenesis of nicotine in COPD, we employed tachypacing with or without H89, KN93 and KB‐R7943 treatment, along with patch clamp experiments and Masson's trichrome staining in control rabbits and rabbits with human leukocyte elastase (0.3 unit/kg)‐induced COPD. Following 20‐Hz tachypacing and isoproterenol treatment, COPD RVOTs had a higher VT incidence than control RVOTs. Nicotine‐treated COPD RVOTs had higher ventricular arrhythmogenesis than non‐treated COPD RVOTs. VTs induced in COPD and nicotine‐treated COPD RVOTs were suppressed by H89, KN93, or KB‐R7943. COPD RVOT myocytes exhibited shorter action potentials than control RVOT myocytes; nicotine‐treated COPD RVOT myocytes exhibited longer action potentials than COPD RVOT myocytes. Both COPD and nicotine‐treated COPD myocytes had smaller L‐type Ca^2+^ currents and larger NCX currents than control RVOT myocytes. Nicotine‐treated COPD RVOT myocytes had larger late Na^+^ currents than control and COPD RVOT myocytes. COPD and nicotine‐treated COPD RVOTs exhibited more fibrosis. Nicotine‐treated COPD RVOTs had the highest level of fibrosis. COPD intensifies RVOT VT through electrical and structural remodelling and Ca^2+^ dysregulation through the activation of PKA, CaMKII and NCX signalling pathways. Nicotine further exacerbates VTs in the rabbit RVOT triggered by COPD.

## 1 Introduction

Cigarette smoke comprises an intricate blend of chemicals, including nicotine and other compounds known for their cardiotoxicity; cigarette smoking thus increases the likelihood of developing ventricular tachycardia (VT) [1]. Moreover, nicotine transdermal patches are widely used for smoking cessation. Nicotine accelerates the heart rate, constricts the coronary arteries and increases the risk of various types of cardiac arrhythmias [1, 2]. Nicotine initially exerts a negative chronotropic effect through vagal stimulation, followed by a positive chronotropic effect resulting from sympathetic stimulation and the release of Ca^2+^ ions [3]. In an animal model, intravenous nicotine administration prompted premature ventricular contractions, sustained VT and ventricular fibrillation [2]. Nicotine has also been demonstrated to directly affect cardiac electrophysiology by inhibiting L‐type Ca^2+^ current (I_Ca‐L_), transient outward K^+^ current (I_to_), rapid delayed rectifier K^+^ current and inward rectifier K^+^ current in cardiomyocytes [4, 5]. Smoking is commonly associated with chronic obstructive pulmonary disease (COPD), which is a significant risk factor for VT [6, 7]. The presence and severity of COPD are correlated with the risk of developing VT [8]. In our previous rabbit model of COPD, we found that COPD induced hypoxaemia, hypercapnia and autonomic dysregulation, in addition to exacerbating cardiac inflammation and oxidative stress [9]. The mechanisms responsible for nicotine‐induced arrhythmogenesis may include nicotine's role in augmenting catecholamine release through the stimulation of nicotinic acetylcholine receptors [1]; however, whether chronic nicotine administration results in cardiac electrical and structural remodelling remains uncertain. Moreover, the arrhythmogenic risk associated with nicotine consumption in individuals at high risk of VT has yet to be conclusively determined.

The right ventricular outflow tract (RVOT), with distinctive electrical features such as action potential duration (APD) dispersions and heightened susceptibility to Ca^2+^ overload, is the most common origin of VT [10, 11, 12]. The RVOT myocardium receives innervation from sympathetic fibres of the ventromedial cardiac nerve [13, 14], and sympathetic nerve stimulation in the RVOT may induce RVOT VT [15]. Consequently, researchers have posited that the dysregulation of Ca^2+^ homeostasis is responsible for the high arrhythmogenic potential in the RVOT. Furthermore, the elevated sympathetic tone observed in individuals who have COPD or who use nicotine may exacerbate the occurrence of RVOT VT. In animal experiments, prolonged intravenous nicotine administration has been reported to induce oxidative stress, inflammation, fibrosis and apoptosis in ventricular myocytes [16, 17, 18]. Therefore, nicotine may increase the incidence of VT in individuals with COPD through its effects on electrical and structural remodelling in the RVOT. Thus, we investigated the arrhythmogenic effects of chronic nicotine administration in an animal model of COPD and explored the underlying mechanisms.

## 2 Methods

### 2.1 COPD Model Establishment and Transdermal Nicotine Treatment

To establish the three experimental groups, we anaesthetised male New Zealand white rabbits, weighing 2.5–3.5 kg, through intramuscular injection using a mixture of Zoletil (10 mg/kg; Virbac, Carros, France) and xylazine (5 mg/kg; Bayer, Leverkusen, Germany). The adequacy of the anaesthetic dosage was verified by the absence of corneal reflexes and motor responses to painful stimuli. The COPD group consisted of rabbits in which emphysema was induced through endotracheal administration of human leukocyte elastase (0.3 unit/kg in 2 mL of saline, Sigma‐Aldrich, St. Louis, MO, USA); the control group rabbits received a vehicle, which has been described in previous studies [9, 19]; and the nicotine‐treated COPD group consisted of COPD rabbits receiving chronic nicotine treatment through transdermal nicotine treatment (1 mg/kg/day, 5 days a week, using Nicotinell, GlaxoSmithKline, Brentford, UK) for 4 weeks, initiated at the same time as the human leukocyte elastase administration for emphysema induction. After 5 days of nicotine treatment, urine was collected from the nicotine‐treated COPD rabbits through a urinary catheter inserted into the bladder through the urethra. Urinary cotinine (the predominant metabolite of nicotine) measurements were conducted at a commercial laboratory (Union Clinical Laboratory, Taipei, Taiwan). All experimental procedures were approved by our institutional animal care and use committee (LAC‐2020‐0431).

### 2.2 Electropharmacological Experiments of RVOT Preparations

The control, COPD and nicotine‐treated COPD rabbits were humanely euthanised using a combination of intramuscular injection with Zoletil (10 mg/kg) and xylazine (5 mg/kg), followed by the administration of an overdose of inhaled isoflurane (5% in oxygen; Panion & BF Biotech, Taoyuan, Taiwan) through a precise vaporiser [20]. The adequacy of the anaesthesia dosage was verified by the absence of corneal reflexes and motor responses to painful stimuli. The heart was swiftly removed through a midline thoracotomy. Subsequently, tissue samples measuring 1 × 1.5 cm^2^ from the RVOT were carefully isolated and superfused with normal Tyrode's solution, which comprised NaCl (137 mM), KCl (4 mM), NaHCO_3_ (15 mM), NaH2PO_4_ (0.5 mM), MgCl_2_ (0.5 mM), CaCl_2_ (2.7 mM) and dextrose (11 mM), at a constant flow rate of 3 mL/min at 37°C. The Tyrode's solution was saturated with a mixture of 97% oxygen and 3% carbon dioxide. The tissue samples were connected to an electrometer (FD223; World Precision Instruments, Sarasota, FL, USA) with a 150‐mg load. The endocardial surface of the tissue preparations was orientated upward, and electrical events were recorded using a Gould 4072 oscilloscope and a Gould TA11 recorder (Gould, Jefferson, OH, USA). The recorded signals were digitised at a resolution of 16 bits and a sampling rate of 125 kHz. For electrical stimulation, a 1‐ms pulse was delivered using a Grass S88 stimulator through a Grass SIU5B stimulus isolation unit (Grass Instruments, Norfolk, MA, USA). VT was defined as the sudden and transient onset and termination of an accelerated spontaneous action potential. VT was categorised as sustained VT (duration ≥ 30 s) and nonsustained VT (duration < 30 s) [21]. RVOT preparations from the control, COPD and nicotine‐treated COPD rabbits were analysed both before and after superfusion with isoproterenol (1 μM) for 3 min, with or without KB‐R7943 (10 μM), KN93 (1 μM) and H89 (10 μM) administration for 15 min to observe the effects of these treatments during 20‐Hz tachypacing for 1 s.

### 2.3 Single RVOT Myocyte Isolation

Single cardiomyocytes from the RVOT were isolated from the three groups of rabbits following humane euthanasia. Per the method of a previous study, isolated hearts were placed on a Langendorff apparatus and superfused in an antegrade manner with oxygenated normal Tyrode's solution at a temperature of 37°C, with pH adjusted to 7.4 by using NaOH [21]. After the hearts were thoroughly cleansed of blood, the perfusate was replaced with an oxygenated Ca^2+^‐free Tyrode's solution that contained 300 units/mL of collagenase type I (Sigma‐Aldrich) and 0.25 units/mL of protease type XIV (Sigma‐Aldrich) for 8–12 min. Following this enzymatic digestion, RVOT tissues, specifically the area within 5 mm below the pulmonary valve, were excised and gently agitated in 50 mL of Ca^2+^‐free oxygenated Tyrode's solution, with pH adjusted to 7.4 using NaOH, until individual RVOT myocytes were successfully isolated. The solution was gradually replaced with normal oxygenated Tyrode's solution, and the cardiomyocytes were allowed to acclimate and stabilise in the bath for a minimum of 30 min before the experiments began.

### 2.4 Ionic Current Measurement

Whole‐cell patch clamp recordings were conducted on single RVOT myocytes using an Axopatch 1D amplifier (Axon Instruments, Foster City, CA, USA) at a controlled temperature of 35°C ± 1°C [21]. Borosilicate glass electrodes with an outer diameter of 1.8 mm and tip resistances of 3–5 MΩ were employed. Action potentials were evoked in single RVOT myocytes lacking spontaneous activity at a paced rate of 1 Hz for 20 beats. The resting membrane potential (RMP) was determined during the period between the previous repolarisation and the onset of the subsequent action potentials. The action potential amplitude (APA) was measured from the RMP to the peak of action potential depolarisation. APDs at 90%, 50% and 20% repolarisation were respectively calculated as APD_90_, APD_50_ and APD_20_. The micropipettes used for measuring the action potentials were filled with a solution containing KCl (20 mM), K‐aspartate (110 mM), MgCl_2_ (1 mM), MgATP (5 mM), HEPES (10 mM), EGTA (0.5 mM), LiGTP (0.1 mM) and Na_2_ phosphocreatine (5 mM), with pH adjusted to 7.2 by using KOH.

To assess the late Na^+^ current (I_Na‐Late_), a step–ramp protocol was employed, involving a transition from −100 mV to +20 mV for 100 ms, followed by a ramp back to −100 mV over 100 ms, per the method of a previous study [21]. These measurements were conducted at room temperature using an external solution consisting of NaCl (130 mM), CsCl (5 mM), MgCl_2_ (1 mM), CaCl_2_ (1 mM), HEPES (10 mM) and glucose (10 mM), with pH adjusted to 7.4 by using NaOH. The micropipettes used were filled with a solution containing CsCl (130 mM), Na_2_ATP (4 mM), MgCl_2_ (1 mM), EGTA (10 mM) and HEPES (5 mM), with pH adjusted to 7.3 by using NaOH. An equilibration period of 5–10 min was adopted to ensure sufficient dialysis and stabilise the cell currents. The I_Na‐Late_ was quantified as the tetrodotoxin (30 μM)‐sensitive portion of the current traces recorded during the voltage ramp back to −100 mV.

The I_Ca‐L_ was measured as an inward current during depolarisation from a holding potential of −50 mV to test potentials ranging from −40 to +60 mV in 10 mV increments, each for a duration of 300 ms, at a frequency of 0.1 Hz. This measurement was conducted using a perforated patch clamp technique with amphotericin B. The external solution contained TEACl (20 mM), CsCl (133 mM), HEPES (10 mM), MgCl_2_ (0.5 mM), CaCl_2_ (1.8 mM) and glucose (10 mM), with pH adjusted to 7.4 by using NaOH. To block the Na^+^ channel and I_to_, tetrodotoxin (10 μM) and 4‐aminopyridine (2 mM) were added to the external solution, respectively. The micropipettes were filled with a solution consisting of CsCl (130 mM), MgCl_2_ (1 mM), Mg_2_ATP (5 mM), HEPES (10 mM), EGTA (10 mM), NaGTP (0.1 mM) and Na_2_ phosphocreatine (5 mM), with pH adjusted to 7.2 by using CsOH, per a previously described method [21].

The I_to_ was examined using a double‐pulse protocol: First, a 30‐ms prepulse, transitioning from −80 to −40 mV, was applied to inactivate the Na^+^ channel. Subsequently, a 300‐ms test pulse, stepping from −40 mV to +60 mV in 10 mV increments, was applied at a frequency of 0.1 Hz. To inhibit the I_Ca‐L_, CdCl_2_ (200 μM) was introduced into the bath solution. The I_to_ was quantified as the difference between the peak outward current and the steady‐state current. The external solution contained NaCl (137 mM), KCl (5.4 mM), HEPES (10 mM), MgCl_2_ (0.5 mM), CaCl_2_ (1.8 mM) and glucose (10 mM), with pH adjusted to 7.4 by using NaOH. The micropipettes were filled with a solution comprising KCl (20 mM), K‐aspartate (110 mM), MgCl_2_ (1 mM), MgATP (5 mM), HEPES (10 mM), EGTA (0.5 mM), NaGTP (0.1 mM) and Na_2_ phosphocreatine (5 mM), with pH adjusted to 7.2 by using KOH [21].

The Na^+^/Ca^2+^ exchanger current (I_NCX_) was determined by subtracting the nickel‐sensitive current recorded in the presence of 10 mM NiCl_2_ from the control current. The recording protocol consisted of 300‐ms pulses spanning from −100 to +100 mV, initiated from a holding potential of −40 mV at a frequency of 0.1 Hz. The external solution used for measuring the I_NCX_ contained NaCl (140 mM), CaCl_2_ (2 mM), MgCl_2_ (1 mM), HEPES (5 mM) and glucose (10 mM), with pH adjusted to 7.4 by using CsOH. The solution was supplemented with strophanthidin (10 mM), nitrendipine (10 mM) and niflumic acid (100 μM). The micropipettes were filled with a solution composed of NaCl (20 mM), CsCl (110 mM), MgCl_2_ (0.4 mM), CaCl_2_ (1.75 mM), tetraethylammonium (20 mM), 1,2‐bis (2‐aminophenoxy) ethane‐N, N, N′, N′‐tetra acetic acid (5 mM), glucose (5 mM), MgATP (5 mM) and HEPES (10 mM), with pH adjusted to 7.25 by using CsOH, as described previously [21].

### 2.5 Histopathology

Histopathological analysis was outsourced to a commercial laboratory (Rapid Science Company, Taichung, Taiwan). Masson's trichrome staining, employed to identify collagen fibres, was applied to the three RVOT tissue preparation groups. Subsequently, the spectral density in the images obtained from each preparation was quantified using Image‐Pro Plus 6.0 software (Media Cybernetics, Rockville, MD, USA).

### 2.6 Statistical Analysis

Continuous variables are presented as mean values with corresponding standard errors. For nominal variables, comparisons were conducted using chi‐square analysis with McNemar's test. To assess differences in variables before and after drug administration among the three groups, two‐way repeated‐measures analysis of variance was employed. Subsequently, the Bonferroni post hoc test was used for pairwise comparisons. *p* < 0.05 indicated statistical significance.

## 3 Results

### 3.1 Electropharmacological Findings in Control, COPD and Nicotine‐Treated COPD RVOTs

Rabbits in the nicotine‐treated COPD group exhibited elevated cotinine levels in urine, with a mean value of 1.09 ± 0.09 μg/mL (*n* = 3).

**FIGURE 1:**
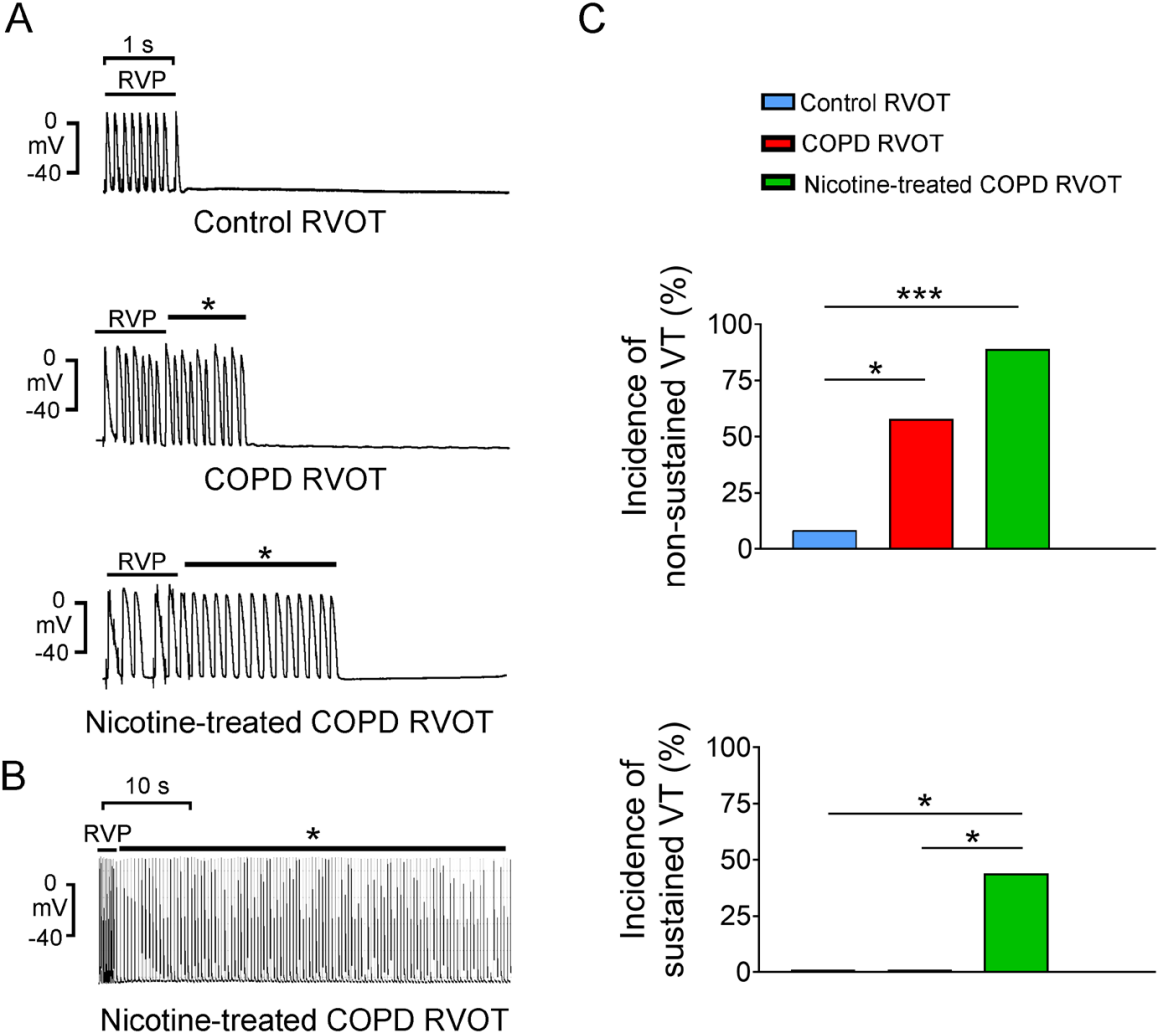
Rapid ventricular pacing (RVP) effects on right ventricular outflow tracts (RVOTs) in control, chronic obstructive pulmonary disease (COPD) and nicotine‐treated COPD groups. (A) The upper panel highlights that RVP (20 Hz) induced no ventricular tachycardia (VT) in control RVOTs. The middle panel illustrates nonsustained VT in COPD RVOTs. The lower panel displays RVP‐induced nonsustained VT in nicotine‐treated COPD RVOTs. (B) Sustained VT is evident in the tracing of nicotine‐treated COPD RVOTs. * indicates nonsustained or sustained VT in panels A and B. (C) The upper and lower panels show the incidence of nonsustained and sustained VT, respectively, induced in control RVOTs (*n* = 12), COPD RVOTs (*n* = 12), and nicotine‐treated COPD RVOTs (*n* = 9). *p < 0.05 and ***p < 0.005.

**FIGURE 2:**
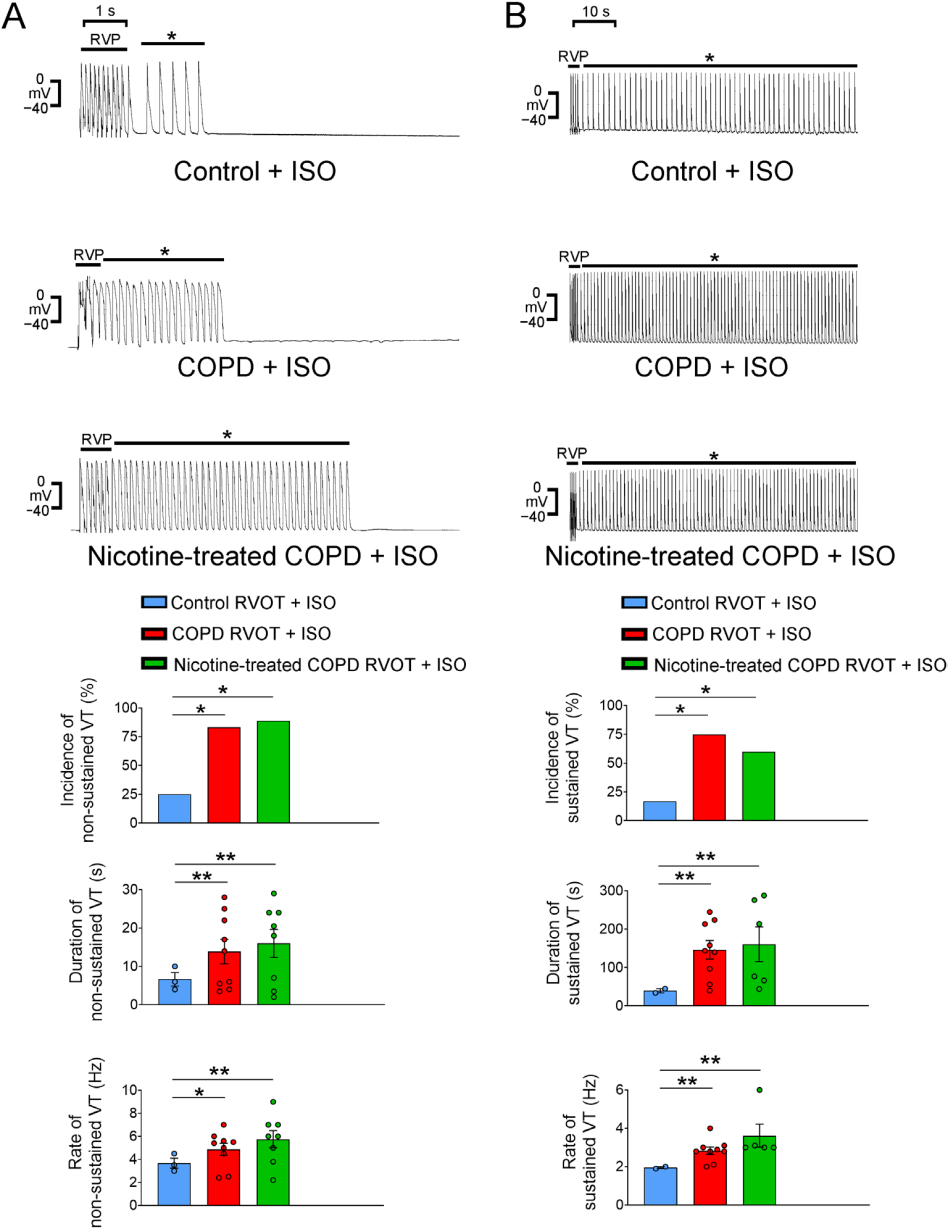
Effect of combined rapid ventricular pacing (RVP, 20 Hz) and isoproterenol (ISO, 1 μM) administration on ventricular tachycardia (VT) occurrence in control, chronic obstructive pulmonary disease (COPD) and nicotine‐treated COPD groups. (A) The upper panels illustrate nonsustained VT induced by RVP and isoproterenol infusion in all three groups. * indicates nonsustained VT. The lower panels illustrate incidence, duration and rate of nonsustained VT induced by RVP and isoproterenol infusion in control RVOTs (*n* = 12), COPD RVOTs (*n* = 12) and nicotine‐treated COPD RVOTs (*n* = 9). (B) The upper panels present sustained VT induced by RVP and isoproterenol infusion in all three groups. * indicates sustained VT. The lower panels display incidence, duration and rate of sustained VT induced by RVP and isoproterenol infusion in different groups. *p < 0.05 and **p < 0.01.

Tachypacing at 20 Hz resulted in a higher occurrence of nonsustained VTs in 7 (58%) of 12 COPD RVOTs (*p* = 0.03) and 8 (89%) of 9 nicotine‐treated COPD RVOTs (*p* = 0.001); in the control RVOTs, only 1 (8.3%) of 12 RVOTs exhibited nonsustained VTs (Figure 1A,C). The incidence of pacing‐induced nonsustained VTs was similar between the COPD RVOTs and nicotine‐treated COPD RVOTs (*p* = 0.296). However, 20‐Hz tachypacing led to sustained VTs in 4 (44%) of 9 nicotine‐treated COPD RVOTs (*p* = 0.045, in comparison with control or COPD RVOTs), whereas none of the 12 control or COPD RVOTs exhibited sustained VTs (Figure 1B,C). Furthermore, following isoproterenol (1 μM) infusion, 20‐Hz tachypacing‐induced 3 (25%) nonsustained VTs and 2 (17%) sustained VTs in 12 control RVOTs, 9 (75%) nonsustained VTs and 9 (75%) sustained VTs in 12 COPD RVOTs and 8 (89%) nonsustained VTs and 6 (75%) sustained VTs in 9 nicotine‐treated COPD RVOTs (Figure 2). The RVOTs from the isoproterenol‐treated COPD and nicotine‐treated COPD groups exhibited a higher incidence of tachypacing‐induced nonsustained and sustained VTs than the control RVOTs. The incidences of combined tachypacing and isoproterenol‐induced nonsustained and sustained VTs were similar between the COPD and nicotine‐treated COPD RVOTs. Furthermore, following combined tachypacing and isoproterenol infusion, both the COPD and nicotine‐treated COPD groups had nonsustained and sustained VTs with an extended duration and a heightened frequency, relative to the control group. Nevertheless, the duration and frequency of pacing‐induced nonsustained and sustained VTs were comparable between the COPD RVOTs and nicotine‐treated COPD RVOTs.

**FIGURE 3:**
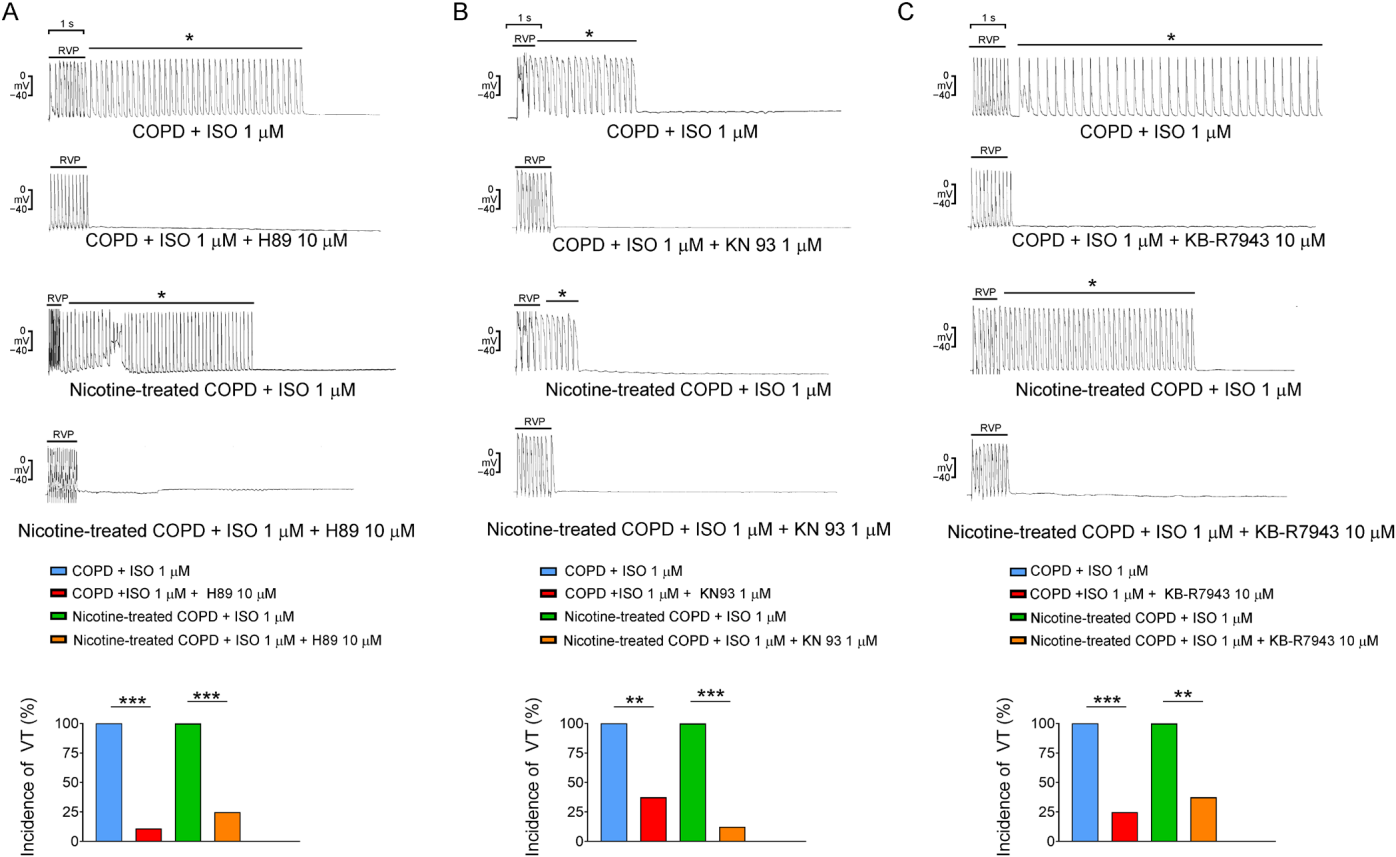
Effects of H89 (10 μM), KN93 (1 μM) and KB‐R7943 (10 μM) treatment on ventricular tachycardia (VT) in right ventricular outflow tract (RVOTs) from chronic obstructive pulmonary disease (COPD) and nicotine‐treated COPD rabbits. The upper panels depict the suppression of nonsustained VTs induced by rapid ventricular pacing (RVP, 20 Hz) and isoproterenol (ISO, 1 μM) infusion upon cotreatment with H89 (panel A), KN93 (panel B) and KB‐R7943 (panel C) in both the COPD and nicotine‐treated COPD groups. * indicates nonsustained or sustained VT. The lower panels illustrate the incidence of nonsustained and sustained VT induced by RVP and isoproterenol infusion, before and after treatment with H89 (Panel A) in both the COPD (*n* = 9) and nicotine‐treated COPD (*n* = 8) groups; KN93 (Panel B) in both the COPD (*n* = 8) and nicotine‐treated COPD (*n* = 8) groups; and KB‐R7943 (Panel C) in both the COPD (*n* = 8) and nicotine‐treated COPD (*n* = 8) groups. **p < 0.01 and ***p < 0.005.

As depicted in Figure 3, H89 (a protein kinase A [PKA] inhibitor, 10 μM) effectively suppressed the nonsustained or sustained VTs induced by combined 20‐Hz tachypacing and isoproterenol treatment in 8 of 9 COPD RVOTs (*p* < 0.001) and 6 of 8 nicotine‐treated COPD RVOTs (*p* = 0.002). Additionally, KN93 (a Ca^2+^/calmodulin‐dependent protein kinase II [CaMKII] inhibitor, 1 μM) suppressed the nonsustained or sustained VTs induced by combined 20‐Hz tachypacing and isoproterenol treatment in 5 of 8 COPD RVOTs (*p* = 0.007) and 7 of 8 nicotine‐treated COPD RVOTs (*p* < 0.001). Finally, KB‐R7954 (an NCX inhibitor, 10 μM) suppressed the nonsustained or sustained VTs induced by the combination of 20‐Hz tachypacing with isoproterenol treatment in 6 of 8 COPD RVOTs (*p* = 0.002) and 5 of 8 nicotine‐treated COPD RVOTs (*p* = 0.007).

### 3.2 Effects of COPD and Nicotine Treatment on Ionic Currents

**FIGURE 4:**
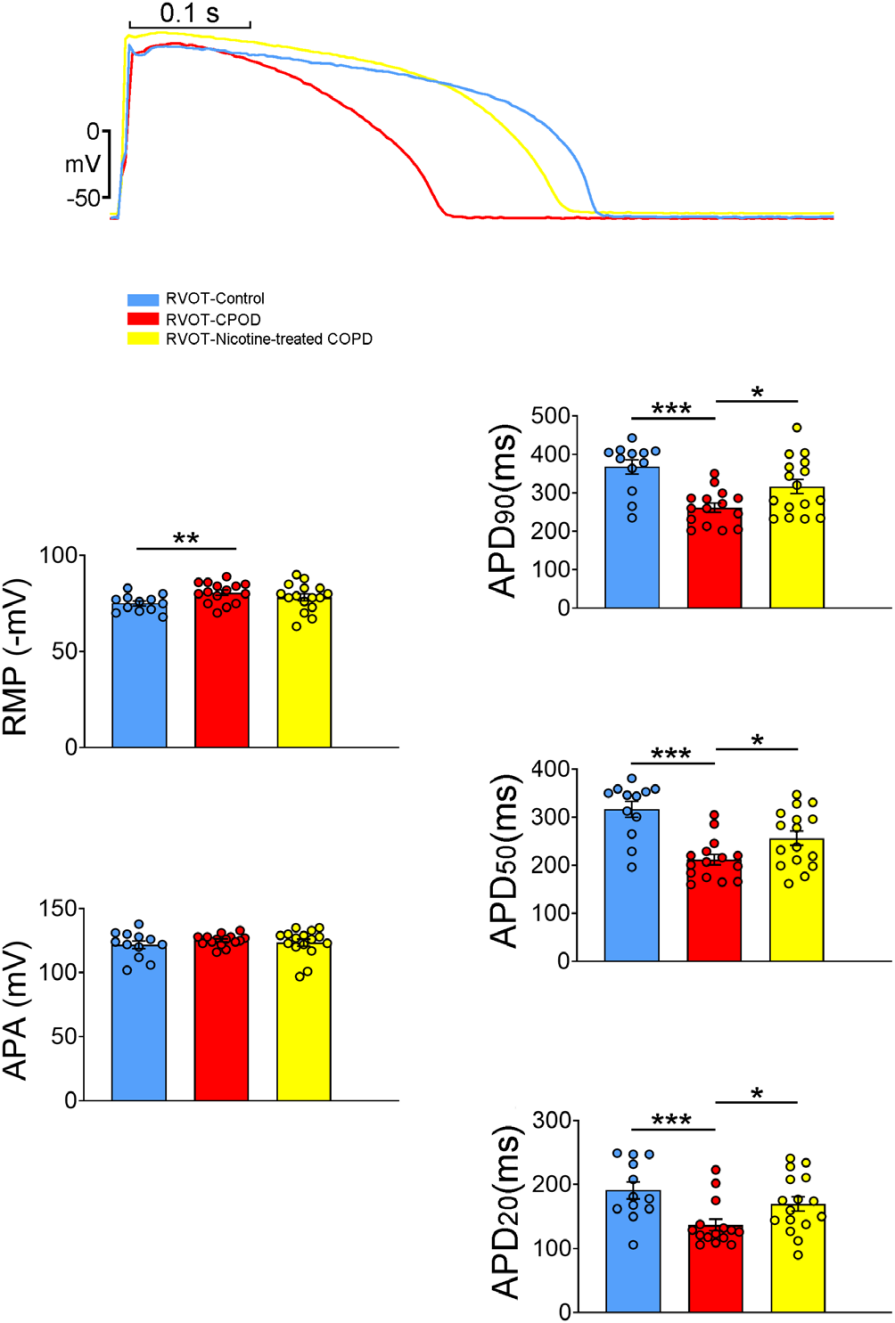
Effects of chronic obstructive pulmonary disease (COPD) and nicotine treatment on action potentials in isolated RVOT myocytes. The upper panel illustrates representative action potential traces from control right ventricular outflow tract (RVOT) myocytes (*n* = 12), COPD RVOT myocytes (*n* = 15) and nicotine‐treated COPD RVOT myocytes (*n* = 16). The lower panels present average data of the action potential parameters in the different groups. APA = action potential amplitude; RMP = resting membrane potential; APD_90_, APD_50_ and APD_20_ = action potential duration at 90%, 50% and 20% repolarisation, respectively. *p < 0.05, **p < 0.01 and ***p < 0.005.

As illustrated in Figure 4, RVOT myocytes from COPD rabbits exhibited a lower RMP and shorter APD at 20%, 50% and 90% repolarisation (APD_20_, APD_50_ and APD_90_) compared to control RVOT myocytes. However, the APA in the COPD RVOT and control RVOT myocytes did not differ significantly. Furthermore, nicotine‐treated COPD RVOT myocytes exhibited longer APD_20_, APD_50_ and APD_90_ than the untreated COPD RVOT myocytes. However, in terms of APA and RMP, the nicotine‐treated COPD RVOT and untreated COPD RVOT myocytes did not differ significantly. Finally, APA, RMP, APD_20_, APD_50_ and APD_90_ were similar between the nicotine‐treated COPD RVOT and control RVOT myocytes.

**FIGURE 5:**
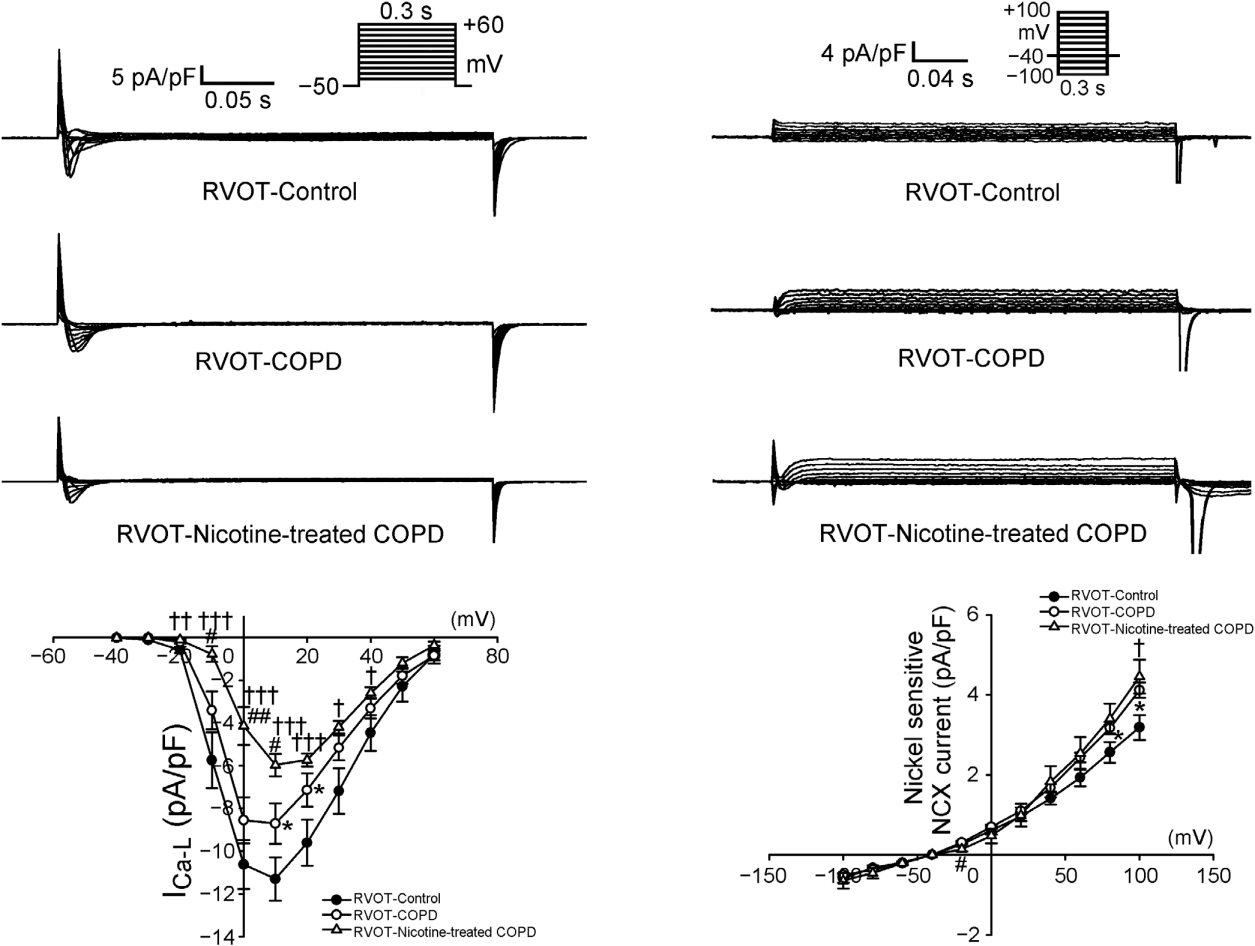
L‐type Ca^2+^ current (I_Ca‐L_) and Na^+^–Ca^2+^ exchanger current (I_NCX_) in right ventricular outflow tract (RVOT) myocytes from control, chronic obstructive pulmonary disease (COPD) and nicotine‐treated COPD rabbits. The left panel, upper part presents the recorded I_Ca‐L_ tracings in control RVOT (*n* = 13), COPD RVOT (*n* = 12) and nicotine‐treated COPD RVOT (*n* = 13) myocytes. The left panel, lower part displays the current–voltage relationship of I_Ca‐L_ within these three groups. *p < 0.05 for when the COPD RVOT myocytes were compared with the control RVOT myocytes; ^†^ *p* < 0.05, ^††^ *p* < 0.01 and ^†††^ *p* < 0.005 for when the nicotine‐treated COPD RVOT myocytes were compared with the control RVOT myocytes; and ^#^ *p* < 0.05 and ^##^ *p* < 0.01 for when nicotine‐treated COPD RVOT myocytes were compared with the COPD RVOT myocytes. The right panel, upper part depicts the recorded I_NCX_ tracings in control RVOT (*n* = 9), COPD RVOT (*n* = 11) and nicotine‐treated COPD RVOT (*n* = 9) myocytes. The lower part presents the current–voltage relationship of I_NCX_ within these three groups. *p < 0.05 for when comparing the COPD RVOT myocytes to the control RVOT myocytes; ^†^ *p* < 0.05 for when the nicotine‐treated COPD RVOT myocytes were compared with the control RVOT myocytes; and ^#^ *p* < 0.05 for when nicotine‐treated COPD RVOT myocytes were compared with the COPD RVOT myocytes.

**FIGURE 6:**
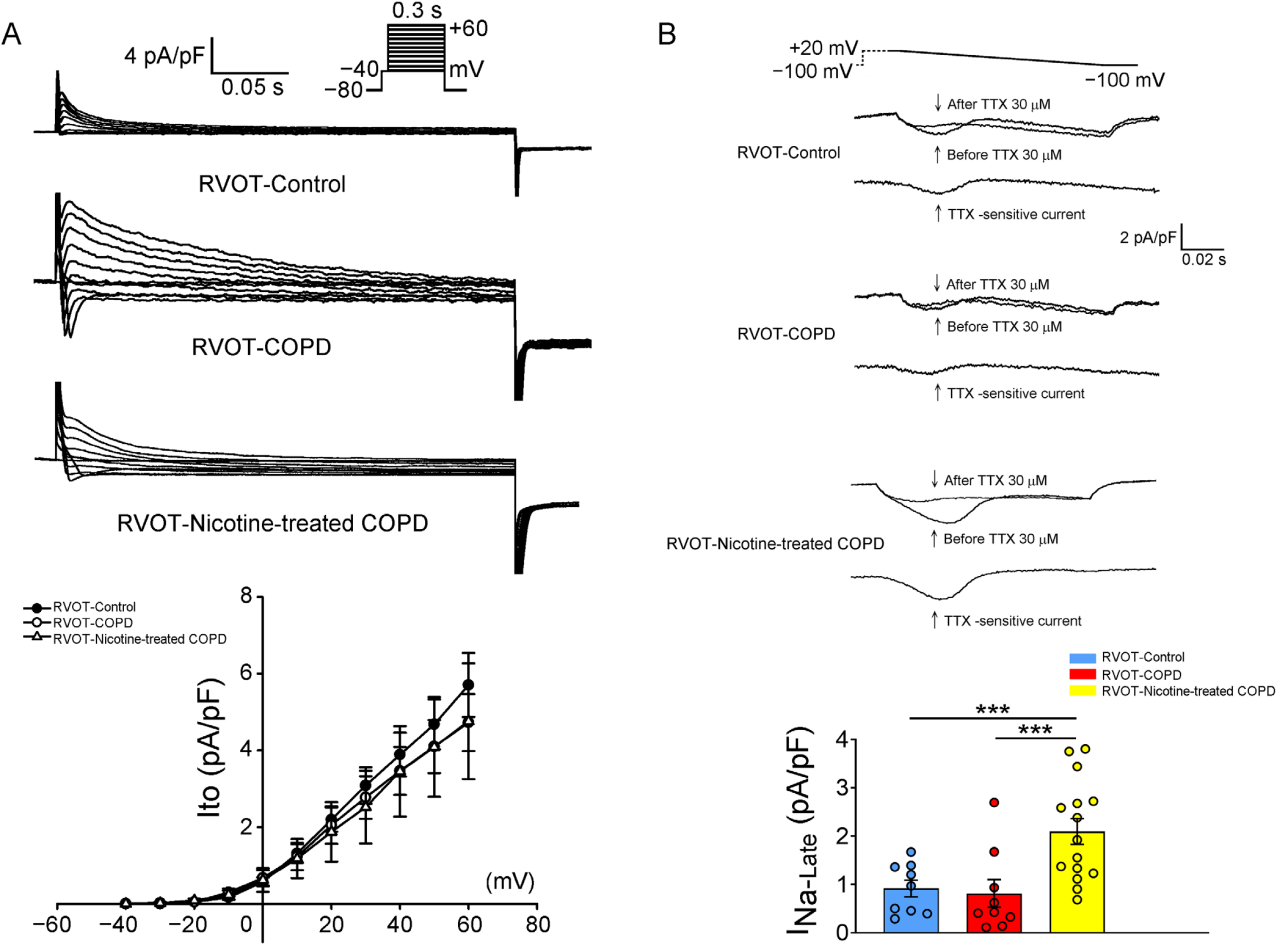
Transient outward K^+^ current (I_to_) and Late Na^+^ current (I_Na‐Late_) in right ventricular outflow tract (RVOT) myocytes from control, chronic obstructive pulmonary disease (COPD) and nicotine‐treated COPD rabbits. (A) The upper panels present the recorded I_to_ tracings in control RVOT (*n* = 16), COPD RVOT (*n* = 9) and nicotine‐treated COPD RVOT myocytes (*n* = 10). The lower panel displays the current–voltage relationship of I_to_ within these three groups. Insets in the current traces indicate the clamp protocols employed. (B) The upper panels present the recorded I_Na‐Late_ tracings in control RVOT (*n* = 9), COPD RVOT (*n* = 9) and nicotine‐treated COPD RVOT (*n* = 15) myocytes. The lower panels display the current–voltage relationship of I_Na‐Late_ in these three groups. ***p < 0.005.

As depicted in Figure 5, both the COPD RVOT and nicotine‐treated COPD RVOT myocytes had a lower I_Ca‐L_ than the control RVOT myocytes. Moreover, nicotine‐treated COPD RVOT myocytes showed a reduction in I_Ca‐L_ compared to COPD RVOT myocytes. Furthermore, both the COPD RVOT myocytes and nicotine‐treated COPD RVOT myocytes had a larger I_NCX_ than the control RVOT myocytes. Nicotine treatment resulted in a reduction in I_NCX_ compared to untreated COPD myocytes. Moreover, the three RVOT groups had a similar I_to_ (Figure 6A). As indicated in Figure 6B, the nicotine‐treated COPD RVOT myocytes had a larger I_Na‐Late_ than the control or COPD RVOT myocytes. However, I_Na‐Late_ did not differ significantly between the control and COPD RVOT myocytes.

### 3.3 Histopathological Study

**FIGURE 7:**
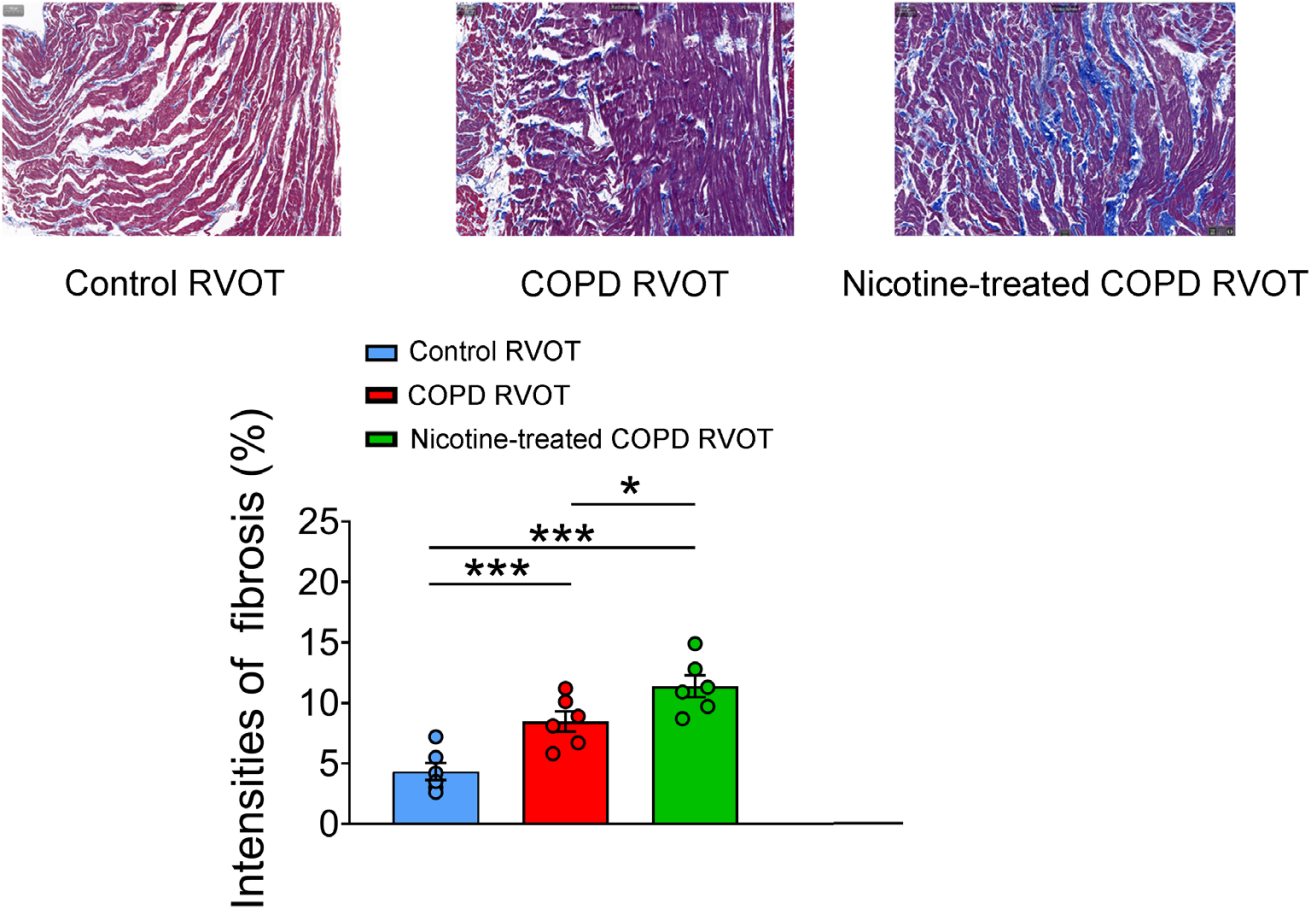
Histopathology of the right ventricular outflow tract (RVOT) from control, chronic obstructive pulmonary disease (COPD) and nicotine‐treated COPD rabbits. The upper panel displays representative Masson‘s trichrome stains. The lower panel presents the average data for the control (*n* = 6), COPD (*n* = 6) and nicotine‐treated COPD (*n* = 6) RVOT myocytes. *p < 0.05 and ***p < 0.005.

Both the COPD RVOTs and nicotine‐treated COPD RVOTs exhibited more pronounced fibrosis than the control RVOTs (Figure 7). Moreover, the nicotine‐treated COPD RVOTs had greater fibrosis than the non‐nicotine‐treated COPD RVOTs.

## 4 Discussion

Our results indicated that both COPD and nicotine‐treated COPD RVOTs had a higher incidence of nonsustained VTs induced by tachypacing at 20 Hz relative to the control RVOTs. Notably, the nicotine‐treated COPD RVOTs displayed a higher incidence of tachypacing‐induced sustained VTs than both the control and COPD RVOTs. These findings suggest that COPD alone can prompt VT in RVOTs, and nicotine treatment exacerbates this propensity in COPD RVOTs. This difference in VT incidence in the different RVOT groups may be due to differences in fibrotic changes in RVOTs, with nicotine‐treated COPD RVOTs exhibiting greater fibrosis than COPD RVOTs. Inflammation is a pivotal pathogenetic mechanism in COPD [22, 23], and inflammatory mediators originating in the lungs can directly affect the heart [24]. Cardiac inflammation leads to progressive structural deterioration, culminating in cardiac fibrosis and extensive cardiac fibrosis induces electromechanical disturbances, thereby establishing a substrate for reentry [25]. In animal models, prolonged intravenous nicotine administration has been demonstrated to induce oxidative stress, inflammation, fibrosis and apoptosis in ventricular cardiomyocytes [16, 17, 18]. Our findings suggest that COPD intensifies inflammation, leading to fibrosis and structural remodelling, which then potentially contribute to RVOT ventricular arrhythmogenesis, a process further exacerbated by nicotine treatment.

Our study also revealed that the combination of tachypacing and isoproterenol treatment can induce sustained VTs both in the COPD and nicotine‐treated COPD RVOTs. Treatment with isoproterenol supported the shift from nonsustained VTs to sustained VTs [26]. These findings suggest that heightened sympathetic activity in COPD and nicotine‐treated COPD RVOTs could amplify RVOT arrhythmogenesis. Additionally, the RVOT exhibits distinctive electrophysiological traits; for example, it has a shorter and more dispersed APD than the left ventricular outflow tract, and it exhibits greater Ca^2+^ dysregulation relative to the right ventricular apex. These differences could contribute to the increased propensity for RVOT arrhythmogenesis [12, 27]. Thus, combined tachypacing and isoproterenol treatment can potentially induce both nonsustained and sustained VTs even in relatively healthy control RVOTs.

Both COPD and nicotine have been linked to chronic sympathetic overactivity [3, 28], which stimulates β1 adrenergic receptors, activating the cAMP‐dependent PKA and CaMKII pathways. These pathways phosphorylate various intracellular Ca^2+^ regulatory proteins, including Ca^2+^ channels, sarcoplasmic/endoplasmic reticulum Ca^2+^‐ATPase 2a, ryanodine receptor, phospholamban and NCX [29, 30, 31, 32], leading to Ca^2+^ dysregulation [33, 34]. In our study, we observed that the VTs induced by combined tachypacing and isoproterenol treatment in COPD or nicotine‐treated COPD RVOTs were mitigated by the administration of H89, KN93, or KB‐R7943. This suggests that the PKA, CaMKII and NCX signalling pathways are implicated in the ventricular arrhythmogenesis observed in COPD or nicotine‐treated COPD RVOTs.

We also found that the COPD RVOTs exhibited a shorter APD than the control RVOTs. However, the nicotine‐treated COPD RVOTs had a longer APD than the COPD RVOTs. These findings imply that both COPD and nicotine treatment may induce electrical remodelling in the RVOT. In our experiments, the COPD RVOT myocytes had a similar I_to_ but a smaller I_Ca‐L_ than the control RVOT myocytes. These observations suggest that the shorter APD in the COPD RVOT myocytes can be attributed to the smaller I_Ca‐L_. Moreover, the nicotine‐treated COPD RVOT myocytes displayed a smaller I_Ca‐L_ but a larger I_Na‐Late_ than both the COPD RVOT and control RVOT myocytes. These findings indicate that nicotine treatment modulates the I_Ca‐L_ and I_Na‐Late_, resulting in the restoration of a shortened APD in nicotine‐treated COPD RVOT myocytes.

In our study, both the COPD RVOT and nicotine‐treated COPD RVOT myocytes had higher I_NCX_ levels than the control RVOT myocytes. I_NCX_ plays a vital role in maintaining the balance of Ca^2+^ during excitation–contraction coupling [35]. Elevated I_NCX_ levels contribute to arrhythmia formation by triggering delayed afterdepolarisation within a spontaneous Ca^2+^ wave, leading to heightened intracellular Ca^2+^ levels during the diastolic phase [36]. Furthermore, we observed that the VTs induced by tachypacing and isoproterenol treatment in the COPD and nicotine‐treated COPD RVOTs were alleviated by the administration of an I_NCX_ inhibitor. These findings strongly support the hypothesis that increased I_NCX_ levels are a key pathological mechanism underlying ventricular arrhythmias associated with COPD and nicotine exposure.

An increased I_Na‐Late_ has been observed in various common pathological conditions such as heart failure, coronary artery disease and left ventricular hypertrophy [37]. I_Na‐Late_ contributes to prolonged depolarisation during the cardiac action potential plateau, creating a foundation for early afterdepolarisations. Elevated I_Na‐Late_ levels can thus prompt Ca^2+^ overload through reverse NCX, leading to spontaneous Ca^2+^ release in the sarcoplasmic reticulum (SR) and subsequent delayed afterdepolarisation [38]. In our study, the COPD RVOT myocytes exhibited a similar I_Na‐Late_ to the control RVOT myocytes. By contrast, the nicotine‐treated COPD RVOT myocytes displayed a larger I_Na‐Late_ than the control and COPD RVOT myocytes. This augmented I_Na‐Late_ in nicotine‐treated COPD RVOT cells may facilitate the onset of ventricular arrhythmias.

Both PKA and CaMKII have been shown to modulate I_Ca‐L_ and I_Na‐Late_, contributing to arrhythmogenesis under pathological conditions. Specifically, adrenergic receptor stimulation significantly increases I_Ca‐L_, mediated by the cAMP/PKA signalling pathway. PKA phosphorylates the α1C subunit of Cav1.2. This phosphorylation increases channel open probability, slows inactivation and increases I_Ca‐L_ amplitude [39]. CaMKII phosphorylates the α1C subunit (at different sites than PKA) and possibly β subunits of the channel, which leads to slower inactivation of I_Ca‐L_ [40]. Similarly, both kinases are implicated in the enhancement of I_Na‐Late_, leading to the prolongation of APD and promoting arrhythmic activity [41]. Our study demonstrated that both I_Ca‐L_ and I_Na‐Late_ changes were increased in nicotine‐treated COPD RVOT myocytes compared to untreated COPD RVOT myocytes. Additionally, the inhibition of PKA and CaMKII effectively suppressed arrhythmias. These findings suggest that nicotine may enhance I_Ca‐L_ and I_Na‐Late_ through PKA and CaMKII signalling pathways, thereby promoting arrhythmogenesis.

PKA and CaMKII signalling pathways have been implicated in the development of cardiac fibrosis. Activation of β_1_‐adrenergic receptors stimulates PKA activity, thereby promoting the proliferation of cardiac fibroblasts [42]. Additionally, the activation of CaMKII in cardiomyocytes by angiotensin II triggers a transcriptional program that drives inflammatory signalling and contributes to the progression of cardiac fibrosis [43]. Although our study was not specifically designed to dissect the signalling pathways leading to fibrosis, further mechanistic studies are needed to definitively establish the causal links between PKA/CaMKII signalling and fibrotic remodelling in this context.

The RVOT is a well‐established arrhythmogenic region, and increased APD dispersion within the RVOT is known to contribute to VT susceptibility [44]. Our studies show that both the COPD RVOTs and nicotine‐treated COPD RVOTs exhibited significantly greater fibrosis compared to control RVOTs. These fibrotic changes create non‐uniform conduction pathways, which may contribute to regional differences in APD. Moreover, changes in I_Ca‐L_ and I_Na‐Late_ may also contribute to regional APD heterogeneity. Furthermore, disrupted SR Ca^2+^ release and reuptake, as well as increased NCX activity, can cause afterdepolarisations and affect APD differently across the RVOT. However, we did not conduct experiments specifically to assess APD dispersion. Currently, there is no direct evidence demonstrating that APD dispersion in the RVOT is increased in COPD models or further enhanced by nicotine treatment.

The present study had some limitations. This study did not perform direct measurements of intracellular Ca^2+^ dynamics, which may provide more definitive mechanistic insight. Further studies that demonstrate the effect of COPD and nicotine treatment on intracellular Ca^2+^ transient recordings and assessments of SR Ca^2+^ release are warranted to validate these findings. Second, our study was primarily focused on the combined effects of COPD and nicotine exposure, which reflects a clinically relevant scenario given the high prevalence of smoking in COPD patients. However, including a nicotine‐treated control group would strengthen the interpretation of nicotine's specific contribution. The absence of a nicotine‐treated control group limits our ability to determine whether nicotine exerts a synergistic effect specifically in the COPD RVOT or if similar effects would also be observed in non‐COPD RVOT tissue. Third, H89, KN93 and KB‐R7943 have known off‐target effects, including potential direct inhibition of ATP‐sensitive K^+^ current, inward rectifier K^+^ current and the rapid component of delayed rectifier K^+^ current—all of which are implicated in arrhythmogenesis [45, 46, 47]. While these agents are widely used as tools to probe the involvement of PKA, CaMKII and NCX, these off‐target effects of H89, KN93 and KB‐R7943 should be considered when interpreting the data. Complementary approaches, such as genetic inhibition or more selective inhibitors, would help validate the specific roles of these pathways and should be considered in future studies. Finally, we intentionally selected 20 Hz pacing to induce arrhythmogenic events in the RVOT, particularly under conditions of COPD and nicotine exposure. At this frequency, pacing can exceed the intrinsic conduction capacity of RVOT tissue, resulting in a loss of 1:1 capture and occasional APD alternans. Although the addition of isoproterenol shortens the refractory period, enabling the myocardium to respond to higher pacing frequencies.

## 5 Conclusions

COPD intensifies ventricular arrhythmogenesis in the RVOT through electrical and structural remodelling, coupled with Ca^2+^ dysregulation facilitated by the activation of the cAMP‐dependent PKA, CaMKII and NCX signalling pathways. Nicotine further exacerbates VTs in the rabbit RVOT triggered by COPD.

## Author Contributions

**Chao‐Shun Chan:** conceptualization (equal), data curation (equal), formal analysis (equal), funding acquisition (equal), writing – original draft (equal). **Feng‐Zhi Lin:** data curation (equal), formal analysis (equal). **Yao‐Chang Chen:** conceptualization (equal), data curation (equal), formal analysis (equal), methodology (equal). **Satoshi Higa:** funding acquisition (equal), writing – review and editing (equal). **Shih‐Ann Chen:** writing – review and editing (equal). **Yi‐Jen Chen:** conceptualization (equal), project administration (equal), supervision (lead), writing – original draft (equal).

## Conflicts of Interest

The authors declare no conflicts of interest.

## Data Availability

Data available on request from the authors.
